# Mothers’ Expectations and Factors Influencing Exclusive Breastfeeding during the First 6 Months

**DOI:** 10.3390/ijerph17010077

**Published:** 2019-12-20

**Authors:** Esmeralda Santacruz-Salas, Isaac Aranda-Reneo, Antonio Segura-Fragoso, Ana Isabel Cobo-Cuenca, José Alberto Laredo-Aguilera, Juan Manuel Carmona-Torres

**Affiliations:** 1 Faculty of Health Sciences, University of Castilla-La Mancha, 45600 Talavera de la Reina, Spain; esmeralda.santacruz@uclm.es (E.S.-S.); antonio.segura@uclm.es (A.S.-F.); josealberto.laredo@uclm.es (J.A.L.-A.); 2 Grupo de Investigación Multidisciplinar en Cuidados (IMCU), Universidad de Castilla-La Mancha, 45071 Toledo, Spain; juanmanuel.carmona@uclm.es; 3 Facultad de Ciencias Sociales, Universidad de Castilla-La Mancha, 45600 Talavera de la Reina, Spain; isaac.aranda@uclm.es; 4 Facultad de Fisioterapia y Enfermería, Universidad de Castilla-La Mancha, 45071 Toledo, Spain; 5 Instituo Maimónides de Investigación Biomédica de Córdoba (IMIBIC), 14004 Córdoba, Spain

**Keywords:** expectations, breastfeeding, exclusive breastfeeding, failure to meet expectations, influencing factors, self-efficacy of breastfeeding

## Abstract

The aims were to determine Spanish women’s expectations about exclusive breastfeeding (EB) and the effect of expectations and other factors on EB during the first 6 months. A prospective cohort study was conducted with 236 participants. Variables were maternal age, marital status, occupation, expectations about breastfeeding, knowledge about breastfeeding, type of delivery, type of feeding, and duration of EB. Data were collected through three personal interviews, at the hospital (before delivery) and in two telephone calls in the first and sixth months postpartum. Average age was 32.3 years (SD = 5.3); average duration of EB was 2.73 months (SD = 2.49). Of 236 women who had decided to breastfeed before birth, 201 (85.2%) offered EB after delivery. Achievement of expectations was most influenced by the decision to continue breastfeeding ‘as long as I can’ (OR: 5.4; CI: 2.0–14.6) and previous experience (OR: 3.2; CI: 1.2–8.5). Knowledge of breastfeeding acquired from relatives (OR: 9.2; CI: 3.0–27.9), caesarean delivery (OR: 4.6; CI: 1.7–12.8) and maternal age (36–40 years old) (OR: 7.5; CI: 1.8–30.9) were associated with failure to achieve EB. Achievement of EB may depend on a woman’s confidence in her ability to do so and on knowledge obtained in the social environment.

## 1. Introduction

The World Health Organization (WHO) and other agencies in charge of child protection and care have confirmed the importance of offering exclusive breastfeeding (EB) for at least 6 months and continuing to offer breastfeeding for the first 2 years of life, supplemented with other foods [1,2,3]. Breastfeeding (BF) is considered the best feeding practice due to the multiple health benefits it provides. BF reduces the incidence of diseases, contributes to the development of a healthy immune system, locomotor system, emotional and psychological state in the newborn, favours better postpartum progress in the mother and prevents certain diseases in the medium and long term [1,3,4,5]. In addition, recent analyses indicate that suboptimal BF practices, including non-EB, contribute to 11.6% mortality in children under 5 years of age. In 2011, this accounted for some 804,000 child deaths [5].

These data highlight the importance of EB. Even with the alarming figures of not providing EB, the current data show rates and prevalence lower than the objectives set out in the recommendations of Healthy People 2020–2025, where the aim is to achieve 50% EB at 6 months [6,7].

The mother’s self-efficacy regarding BF plays an important role in the initiation and progression of the recommended minimum period [8,9]. It has been shown that mothers with high self-efficacy in BF experience fewer problems both starting and maintaining BF [10].

Between 90% and 100% of women choose to offer BF as a way to feed their babies [11,12,13], but figures observed at 6 months (20–25%) are far below targets [11,12,13]. Several factors are observed to frustrate the expectations of mothers in their BF purpose. For this reason, in this study, we set ourselves the main objective of understanding mothers’ expectations regarding the duration of BF. Secondary objectives were a) to identify the elements that frustrate women’s expectations and b) to study the effect that mothers’ expectations at the time of delivery have on the achievement of EB in the first 6 months of the child’s life.

## 2. Materials and Methods

### 2.1. Study Design and Participants

We designed and performed a prospective cohort study with a Spanish population. The follow-up period was the first 6 months of the child’s life. In Spain, health services are largely delivered by public bodies and provide healthcare for the entire population. Free check-ups are included for the mother and her infant after childbirth, among other healthcare services.

In addition to the interview with the mother during the hospitalization period (visit 0), the data from the follow-up period for this study were validated by medical record and collected via telephone interviews with the women who participated in the study: one during the health visit to the newborn at 1 month postpartum (visit 1) and another at 6 months (visit 2). A period of 6 months EB is recommended by the WHO.

The inclusion criteria were that the mothers had given birth to a healthy newborn between 37 and 42 weeks of gestation and weighing more than 2.5 kg. The exclusion criteria were: mothers of children who were being adopted, newborns who were admitted to the Neonatal Service for any pathology, newborns who tested positive during neonatal screening, mothers with multiple gestations and mothers who required hospitalization due to postpartum complications.

This hospital has not been certified as baby friendly. All mothers were invited to participate in the study before the birth of their child and after signing to indicate their informed consent about the study’s objectives. The study was evaluated by the Ethics Committee of Clinical Reference Research [Ethics Committee of Toledo Hospital Complex; number 74; date: 6/06/2014].

### 2.2. Sample

**Figure 1:**
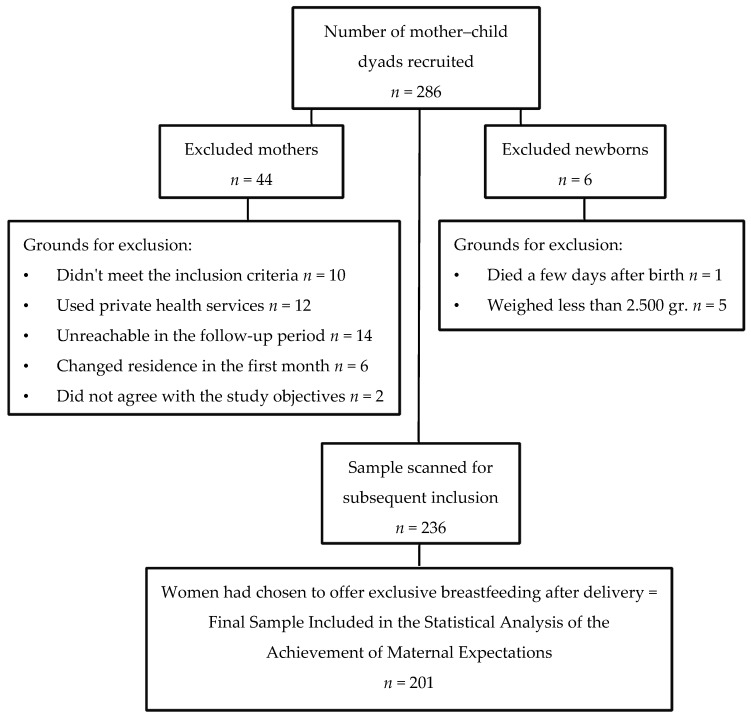
Flowchart of the sample selection process.

The selection of women who participated in the study was carried out consecutively among all the mothers who were admitted to give birth between June 13 and August 10, 2014. Losses at follow-up and exclusions are shown in the flowchart in Figure 1.

The incorporation of participants was carried out by personnel unaware of the objectives of the study and who did not subsequently participate in any analysis of the data. To ensure the anonymity of women during follow-up, a participant code was created.

### 2.3. Outcome Measures

Regarding the different types of feeding of the child registered, we made distinctions between:

Exclusive breastfeeding (EB), which was defined as feeding where the only milk consumed was of human origin, taken directly from the breast or bottle.

Mixed breastfeeding (MB), which was defined as the consumption of human milk along with other types of milk or non-human formula.

Formula feeding (FF): consumption of exclusively formula milk

The initial survey (visit 0) collected information about the general characteristics of the participants in the study, including working and sociodemographic conditions, clinical characteristics of pregnancy, childbirth, postpartum elements that could be related to the initiation and/or maintenance of BF, and the expectations of women about BF. To obtain specific information regarding achievement the maternal expectations (dependent variable) in relation to EB, a question was included about how long she intended to maintain EB. The response options were: 1 month, 3 months, between 3 and 6 months, ‘as long as I can’ and ‘as long as the baby wants it’. Table 1 shows the options that determine whether maternal expectations were achieved or not.

The telephone interviews that were carried out during the follow-up period (visit 1 and visit 2) allowed us to quantify the degree to which the expectations of the women who participated in the study were fulfilled, with respect to their intention to offer EB at the basal moment (immediately postpartum). To do this, they were asked what type of food they were using in month 1 and in month 6; if this differed from the type of food marked at baseline, they were asked why they decided to make a change in the type of feeding of the child. Among the reasons they indicated for early abandonment of EB, before 6 months, and therefore introduced FF or solid foods, a distinction was made between (i) reasons related to the mother (those mothers who reported having problems related to BF or work reasons), (ii) reasons related to the child (the child ‘did not gain weight’ or the mother believed that the baby ‘was left hungry’ after feeding), and (iii) reasons related to the social environment (‘by recommendation of a health professional’, a feeling of ‘psychological pressure’ from family or close friends).

### 2.4. Statistical Analysis

When mothers were unable to fulfil their own expectations, an analysis was carried out to determine the cause (maternal, baby or social environment). Non-achievement of expectations was determined using logistic regression models. The dependent variable was the achievement of expectations: ‘0′ if they were not achieved and ‘1′ if they were achieved. The reasons indicated by the women as causes for a change in the type of feeding during the follow-up period were included as independent variables. The following control variables were included in the models: the sex of the infant, whether the mother had family support in the upbringing of the child, age of the mother, recommendations of a healthcare provider for the abandonment of EB, social or family pressure, and receipt of knowledge about EB through support groups for EB, professionals or independent research by the mother or by relatives.

The data were analysed using the statistical software IBM-SPSS v 23.0.0.0 (IBM Corp., Armonk, NY, USA). All data were tabulated using the absolute value and percentage with a 95% confidence interval.

## 3. Results

Of the 236 women, 219 (92.8%) had decided, prior to delivery, the type of feeding they wanted to offer their infant; 201 women (85.2%) had decided to offer EB after delivery. Therefore, the statistical analysis on the achievement or frustration of expectations was done for 201 women. When the women left the hospital, only 154 (65.3%) declared that they offered EB.

The average age of the mothers was 32.3 years (SD: 5.3; CI 95%: 19–45).

In the 236 women surveyed, the mean duration for the different types of feeding was 2.73 months for EB, 1.46 months for MB and 2.03 months for FF.

The sociodemographic characteristics, according to achievement of the mothers’ expectations, are shown in Table 2.

Description of the mothers’ different expectations regarding the time they wanted to maintain EB is shown in Table 3.

The answers that the mothers offered to justify the early abandonment of EB were not exclusive, so they could mention one or more reasons. Grouping the information, 49 of 93 (52%) were for maternal reasons, the same number for reasons related to the baby, and 67 of 69 (97.1%) for reasons related to the social environment.

Looking at the results in Table 4, influential conditions in the achievement of maternal expectations, we can conclude that when women decided to offer EB and maintain it ‘as long as I can’, the odds ratio of achieving expectations was five times higher than for women who set different expectations. In women who had previous experience of offering EB to other children, the odds ratio of achieving expectations was also much higher than for women who did not have previous experience. Finally, when the child received bottles of water or oral rehydration solutions (ORS) in the hospital, a lower odds ratio of compliance with maternal expectations was observed.

When studying the effect of the different sociodemographic and clinical variables on the non-achievement of maternal expectations, due to maternal, baby or social environment conditions, it was observed that certain maternal conditions are capable of negatively influencing the initial goals or expectations of these women. All other variables introduced in the initial statistical model did not offer statistical significance; the final model with the influential variables is shown in Table 5.

Among the variables included in the logistic regression analysis, it should be noted that women between 36 and 40 years were 7.5 times more likely to not see their expectations achieved than those under 25 years. Caesarean delivery increased the chances of non-compliance with women’s expectations by almost five times. It should also be noted that when mothers reported having been informed about BF through family members and the social environment, they were likely to not achieve their expectations.

## 4. Discussion

The present study identifies the expectations that mothers have regarding BF and the duration they wish to provide this type of feeding to their babies. It is observed that these expectations are affected by different elements during the first 6 months of their infant’s life.

Most mothers (91.8%; 201 of 219) had expectations of feeding their child with EB. This is a high percentage of intention; however, following 3 days of hospital stay, only 65.25% (154 of 201) left the hospital and managed to maintain EB. If we observe the prevalence of compliance with their initial expectations or objectives with respect to the time EB is maintained, more than half of the mothers did not achieve EB. The high rate of EB abandonment during the hospital stay should be considered by health professionals. These first moments involve a process of adaptation and skills development by the mother, in addition to possible problems that may appear, such as chest and nipple pain and mastitis, among others [14]. There are factors, therefore, that have a negative influence in a short period of time, so it is even more necessary to involve said professionals in achievement of the maternal objectives and in the start and maintenance of EB.

A relevant condition in the achievement of the objectives set is the previous experience of the women in having offered EB, for either less than or more than 6 months. Regarding new mothers, it may be that expectations regarding BF are based on a vision of a natural process that will be pleasant and not cause any additional difficulties. Therefore, when difficulties arise, mothers feel that they are not prepared to overcome them and may become frustrated when the problems do not resolve themselves with maternal instinct alone [15]. To this, we can add other possible factors, such as ignorance of the multiple benefits EB provides. Studies show that more than 60% of mothers who did not breastfeed reported that formula was equal to or better than breast milk [16]. There are other factors, such as difficulties in solving problems with BF, recommendations for unnecessary supplementation and pressures from the immediate social environment. All of these can be reasons for early abandonment and causes of frustration of the initial expectations held by mothers.

Studies show that offering water or ORS to the child after birth interferes negatively with the maintenance of EB [17,18,19]. In our study, in addition, we can conclude that this practice is an unfavourable condition for achieving the expectations of EB maintenance which the mother had initially raised. The chances of not achieving her goal are increased by 60% when the mother offers this supplement to the newborn’s diet.

Multiple studies have determined different elements, both intrinsic and external to women, that influence the initiation and maintenance of EB. These include the characteristics of the mother, the family, the child and the health system itself [19]. Based on this classification, the conditions that influence the non-achievement of maternal expectations have been analysed.

Regarding the factors inherent to women, maternal age is identified in several studies as a condition that influences the maintenance of EB [13,19,20]. In our case, age also influenced non-achievement of the objectives established in terms of the time that EB is maintained, specifically in women over 35 years, as in the work of Miñano Mercadojainor [20].

Among the other factors that negatively influence the achievement of maternal goals are caesarean section as a type of delivery. This practice is already recognized by several authors as a praxis capable of having a negative impact on the initiation and maintenance of BF.

On the other hand, the importance of psychological factors in the initiation and duration of BF has been proven. Among these psychological elements are the expectations that each woman has set, even sometimes before delivery [21,22]. These expectations in turn are related to the self-efficacy of the woman in BF, that is, the perception she has of their capacity to offer the breast [21]. Tully et al. found that mothers who are motivated to start BF by the benefits that it brings for them have better self-efficacy indices for EB during the hospital period, compared to those that only indicate motivation for the benefits for the baby [10]. These conclusions seem to coincide with the results of our study. Those women who indicated keeping EB ‘as long as I can’ are the ones who obtained the best results of compliance (almost 70%). In addition, this condition increases the chances of achieving expectations by almost five times. This again confirms the conclusions of Muñoz Cruz et al. (2017) on the importance of the self-efficacy of BF [21]. On the other hand, the higher percentage of women (97%) who did not achieve their initial expectations about the time they wanted to offer EB were those who indicated ‘environmental social factors’, followed by those who indicated reasons related to the baby or themselves (52%).

Social environmental factors refer to the recommendations of a healthcare professional to introduce another type of food different from breast milk and/or the psychological pressure to which a woman is subjected, including anxiety, doubts, problem solving, advice, etc. Therefore, the motivation and conviction of the mother, without negative external influences, seems to be revealed as a very important factor for the initiation and maintenance of EB. In our work, when the woman reported having obtained knowledge about BF, from BF support groups or family and social environment, her expectations were not achieved to a greater extent. However, scientific evidence shows that the influence of the social environment can be beneficial [23]. It has been proven that a social environment of close friends and relatives who provide quality support with regard to BF helps women to achieve better results in BF [24,25,26,27,28]. Other studies have demonstrated that the intervention of health professionals leads to a significant difference in the initiation and continuation of BF [24,27,29]. Therefore, a common approach throughout society is necessary regarding information and training about BF. Health professionals and, in particular, the midwife and/or reference nurse, are key figures that can help achieve the initial objectives of these mothers, offering their support and knowledge for the benefit of the BF.

The main limitations of our work are the loss of follow-up information on some women in the initial sample collected. In addition, the collection of information was done using a recall method with its consequent bias. Also, at the time of the study, there was no validated questionnaire for the Spanish population. However, the strength of this study is based on the fact that it is a cross-sectional study with data collection from the same women over a period of 6 months.

## 5. Conclusions

This study describes a prospective cohort study of Spanish women to understand why new mothers do not achieve their goals for EB duration. The topic is novel and of great interest to students of BF and lactation.

The expectations of women regarding the desired duration of maintaining EB are not achieved due to personal factors, wrong practices and the influence of the social environment. The greatest influence on the continuation of EB is the mother’s expectation of BF ‘for as long as I can’.

The age of the mother and the family environment of the woman play a very important role in the achievement of expectations about BF duration that women form when they decide to have a child. The impact of the type of delivery in attaining the objectives proposed by the WHO regarding BF must also be considered.

## Figures and Tables

**Table 1:** Conditions for not achieving initial expectations.

Time the Mothers Expected to Offer EB ^1^	Expectations Were Not Achieved When...
1 month	They offered <30 days EB
3 months	They offered <90 days EB
Between 3 and 6 months	They maintained EB between ≤9 and >180 days
As long as I can	They offered <179 days EB and fulfilled one or both of the following conditions for abandonment:
	● Work-related reasons
	● Problem with BF
As long as the baby wants it	They offered <179 days EB and fulfilled one or both of the following conditions for abandonment:
	● Belief that the baby is left hungry
	● The baby is not gaining weight

^1^ EB = exclusive breastfeeding.

**Table 2:** Prevalence of compliance with maternal expectations according to independent sociodemographic and clinical variables (*n* = 201).

Variables		Did Not Achieve Expectations	Achieved Expectations	*p* *
		*n* (%)	*n* (%)	
Age of the mother				
	Up to 30 years	24 (45.3)	29 (54.7)	0.39
	More than 30 years	77 (52)	71 (48)	
Nationality				
	Spanish	89 (52)	82 (48)	0.30
	Foreign	13 (41.9)	18 (58.1)	
Marital status				
	Married	65 (51.6)	61 (48.4)	0.54
	Single	35 (49.3)	36 (50.7)	
	Divorced/separated	1 (25)	3 (75)	
	Widow	0 (0)	0 (0)	
	Without a partner	1 (100)	0 (0)	
Work situation				
	Self employed	8 (61.5)	5 (38.5)	0.09
	Permanent employment	42 (60)	28 (40)	
	Temporary employment	5 (31.2)	11 (68.8)	
	Unemployed	47 (45.6)	56 (54.4)	
Education level				
	Primary school	24 (44.4)	30 (55.6)	0.29
	Secondary school or higher	78 (52.7)	70 (47.3)	
Type of delivery				
	Vaginal delivery	68 (50)	68 (50)	0.59
	Instrumental delivery	10 (62.5)	6 (37.5)	
	Caesarean section	24 (48)	26 (52)	
Total		101 (50.5)	100 (49.5)	201 (100)

* *p* < 0.05

**Table 3:** Prevalence of women’s expectations regarding the desired maintenance period of EB. ^1.^

Amount of Time Mother Expected to Provide EB Prior to Birth	Did Not Achieve Expectations		Achieved Expectations	
	*n* (%)	CI 95% ^2^	*n* (%)	CI 95%
1 month	2 (100)		0 (0.0)	
3 months	1 (50)	(1.3–90.6)	1 (50.0)	(1.3–90.6)
3–6 months	58 (72.5)	(61.4–81.1)	22 (27.5)	(18.1–38.2)
As long as the baby wants it	11 (52.4)	(32.2–73.2)	10 (47.6)	(24.4–65.5)
As long as I can	29 (30.2)	(21.3–40.0)	67 (69.8)	(59.6–7.1)
Total	101 (50.2)	(43.4–57.3)	100 (49.8)	(42.4–56.4)

^1^ EB = exclusive breastfeeding. ^2^ CI = confidence interval.

**Table 4:** Influential conditions in the achievement of maternal expectations.

Influential Variables	OR ^1^	CI 95% ^2^
**Time chosen to maintain EB**		
Chose to continue EB ^3^ for different periods of time	REFERENCE	
Chose to continue EB ‘as long as I can’	5.4	(2.0–14.6)
**Offer of water or ORS**		
They offered no bottles of water or ORS ^4^	REFERENCE	
Offered bottles of water or ORS	0.4	(0.1–0.9)
**Offer of water or ORS**		
Had not offered EB to other children	REFERENCE	
Had offered EB to other children < 6 months	3.2	(1.2–8.5)
Had offered EB to other children ≥ 6 months	4.8	(1.5–14.9)

^1^ OR = odds ratio. ^2^ CI = confidence interval. ^3^ EB = exclusive breastfeeding. ^4^ ORS = oral rehydration solutions.

**Table 5:** Influential conditions to not achieve the expectations of exclusive breastfeeding.

Maternal Conditions	Expectations Not Achieved		
	*n* (%)	OR ^1^	CI 95% ^2^
**Maternal age**			
Maternal age up to 25 years	12 (60)	REFERENCE	
Maternal age between 26 and 30 years	15 (55)	1.8	(0.4–7.5)
Maternal age between 31 and 35 years	47 (61)	1.9	(0.6–6.9)
Maternal age between 36 and 40 years	35 (77.8)	7.5	(1.8–30.9)
Maternal age greater than 40 years	6 (66.7)	2.2	(0.3–15.3)
**Type of delivery**			
Natural birth	70 (57.9)	REFERENCE	
Birth with instruments	8 (57.1)	0.5	(0.1–1.9)
Birth through caesarean section	37 (84.1)	4.6	(1.7–12.8)
**Knowledge of BF**			
Not having knowledge of BF ^3^ by…	REFERENCE		
Knowledge of BF through relatives or the environment	37 (88.1)	9.2	(3.0–27.9)
Knowledge of BF through BF support groups	57 (73.1)	2.4	(1.1–5.1)

^1^ OR = odds ratio. ^2^ CI = confidence interval. ^3^ EB = exclusive breastfeeding.
